# The Effects of Brief Heat During Early Booting on Reproductive, Developmental, and Chlorophyll Physiological Performance in Common Wheat (*Triticum aestivum L.)*

**DOI:** 10.3389/fpls.2022.886541

**Published:** 2022-05-16

**Authors:** Jiemeng Xu, Claudia Lowe, Sergio G. Hernandez-Leon, Susanne Dreisigacker, Matthew P. Reynolds, Elisa M. Valenzuela-Soto, Matthew J. Paul, Sigrid Heuer

**Affiliations:** ^1^Plant Science Department, Rothamsted Research, Harpenden, United Kingdom; ^2^Centro de Investigación en Alimentación y Desarrollo A.C., Carretera Gustavo Enrique Aztiazarán Rosas, Hermosillo, Mexico; ^3^International Maize and Wheat Improvement Center (CIMMYT), Texcoco, Mexico; ^4^Pre-Breeding Department, National Institute of Agricultual Botany (NIAB), Cambridge, United Kingdom

**Keywords:** heat stress, booting, pollen viability, tillering, SPAD and Fv/Fm, wheat

## Abstract

Rising temperatures due to climate change threaten agricultural crop productivity. As a cool-season crop, wheat is heat-sensitive, but often exposed to high temperatures during the cultivation period. In the current study, a bread wheat panel of spring wheat genotypes, including putatively heat-tolerant Australian and CIMMYT genotypes, was exposed to a 5-day mild (34°C/28°C, day/night) or extreme (37°C/27°C) heat stress during the sensitive pollen developmental stage. Worsening effects on anther morphology were observed, as heat stress increased from mild to extreme. Even under mild heat, a significant decrease in pollen viability and number of grains per spike from primary spike was observed compared with the control (21°C/15°C), with Sunstar and two CIMMYT breeding lines performing well. A heat-specific positive correlation between the two traits indicates the important role of pollen fertility for grain setting. Interestingly, both mild and extreme heat induced development of new tillers after the heat stress, providing an alternative sink for accumulated photosynthates and significantly contributing to the final yield. Measurements of flag leaf maximum potential quantum efficiency of photosystem II (Fv/Fm) showed an initial inhibition after the heat treatment, followed by a full recovery within a few days. Despite this, model fitting using chlorophyll soil plant analysis development (SPAD) measurements showed an earlier onset or faster senescence rate under heat stress. The data presented here provide interesting entry points for further research into pollen fertility, tillering dynamics, and leaf senescence under heat. The identified heat-tolerant wheat genotypes can be used to dissect the underlying mechanisms and breed climate-resilient wheat.

## Introduction

Wheat is one of the most important crops for human consumption, grown on 220 million hectares with a total production of 760 million tons in 2020 (FAOSTAT). In scenarios of climate change, wheat plants are prone to be exposed to warmer and more variable temperatures (Trnka et al., 2014). Beyond a physiological threshold, high temperatures cause stress and impair plant growth and development. Both historical data and future predictions have revealed the negative effects of heat on wheat productivity at the global and regional scale (Liu et al., 2016; Zampieri et al., 2017; Pequeno et al., 2021). Therefore, it is crucial to identify and breed heat-adapted varieties to sustain wheat production and ensure food security.

In nature, the adverse effects of heat stress on plants can be variable depending on the intensity, duration, and developmental stage (Stone and Nicolas, 1995; Yeh et al., 2012). Most of the heat-related studies in wheat have been field-based and used late sowing to expose plants to high temperatures during the flowering and grain filling stages; however, short episodes of heat during earlier reproductive stages can also cause significant damage (Zampieri et al., 2017). Indeed, anther and pollen development are considered to be the stages most vulnerable to heat stress (Zinn et al., 2010; Rieu et al., 2017). Stage-specific treatments have found that wheat is particularly sensitive to heat around 8 days before anthesis, which coincides with the early meiosis to tetrad stage of pollen development (Saini and Aspinall, 1982; Prasad and Djanaguiraman, 2014). Because pollen development occurs during the booting stage, while spikes are still inside the developing pseudostem in wheat, the length of the auricles between the flag leaf and the penultimate leaf (referred as auricle interval length, AIL) has been used as a proxy for pollen development. AIL between 3 and 6 cm has been associated with this sensitive stage (Bokshi et al., 2021; Erena et al., 2021). Brief heat exposure during this sensitive period resulted in abnormal meiosis behavior (Omidi et al., 2014; Draeger and Moore, 2017) and a significant reduction in pollen fertility (Prasad and Djanaguiraman, 2014; Begcy et al., 2018; Browne et al., 2021). A few studies have examined the natural variation in pollen viability under heat stress and its association with yield, as booting usually occurs during the cooler time of the cropping season and it is difficult to apply precise stage-specific heat stress (Bheemanahalli et al., 2019; Bokshi et al., 2021). However, considering a warmer and increasingly erratic climate, this area warrants further investigation.

Spike number is one of the main components in determining wheat yield; it is highly variable and responsive to the environmental factors (Slafer et al., 2014). Interestingly, contrasting responses of spike number under heat stress have been reported. When exposed to continuous high temperatures during the terminal flowering and grain filling stages, spike formation and tillering were always reduced (Cai et al., 2016; Sharma et al., 2016; Dwivedi et al., 2017; Kumar et al., 2021). In contrast, after a short episode of heat stress during earlier developmental stages, spike numbers increased (Bányai et al., 2014; Chavan et al., 2019; Hütsch et al., 2019). Enhanced spike formation after early heat stress is surprising, but the underlying tillering dynamics and impact on final yield have not been discussed.

In addition to the effects on pollen fertility and spike number, heat-induced yield loss has also been ascribed to accelerated leaf senescence, shortening the duration of grain filling (Cossani and Reynolds, 2012; Pinto et al., 2016; Shirdelmoghanloo et al., 2016; Bergkamp et al., 2018; Sade et al., 2018). As indicators of senescence, chlorophyll soil plant analysis development (SPAD) (chlorophyll content index) (Richardson et al., 2002) and Fv/Fm (the maximum potential quantum yield of photosystem II) (Murchie and Lawson, 2013) have been widely used to evaluate this trait. Under terminal heat, SPAD and Fv/Fm were often reduced in leaf tissue during senescence and were closely related to yield-contributing traits, such as thousand grain weight (Talukder et al., 2014; Hassan et al., 2018; Mirosavljević et al., 2021; Touzy et al., 2022). Studies for the genetic analysis of these leaf senescence related traits are also available (Azam et al., 2015; Bhusal et al., 2018; Touzy et al., 2022). Nevertheless, time course measurements of SPAD and Fv/Fm, which enable model fitting and senescence parameter prediction, have rarely been captured in wheat under heat stress (Pinto et al., 2016; Šebela et al., 2020; Touzy et al., 2022), especially after brief heat during the early reproductive stage. In the present study, a wheat heat panel, including putatively heat-tolerant Australian and CIMMYT-nominated spring wheat genotypes, was exposed to a 5-day extreme (37°C/27°C) (Erena, 2018) or mild (34°C/28°C) heat stress during the early pollen developmental stage and analyzed for the effects on (i) pollen viability and seed set; (ii) spike formation and underlying tillering dynamics; (iii) leaf senescence measured with SPAD and Fv/Fm; and (iv) relationships among the reproductive, developmental, physiological, and yield-related traits.

## Materials and Methods

### Plant Material

In this study, three greenhouse experiments (i.e., Exp1, Exp2, and Exp3) were conducted during 2020–2021 at the controlled environment and glasshouse facilities at Rothamsted Research, Harpenden, United Kingdom (51.8094°N, 0.3561°W). For Exp1 and Exp2, the same set of 14 wheat genotypes was used, and 22 lines (seven overlapping with Exp1 and Exp2) were grown in Exp3 (refer to Supplementary Table 1 for genotype details). These lines are putatively heat-tolerant elite spring varieties (Sunstar, Sokoll, and Waagaan) parental lines and their pre-breeding materials (Cossani and Reynolds, 2015; Erena, 2018), as well as two lines from the United Kingdom included as controls.

### Experimental Design and Heat Stress Treatment

#### Exp1

This experiment was conducted between June and September of 2020 and followed a split randomized complete block design (split RCBD) with four blocks/biological replicates. Fourteen genotypes (Supplementary Table 1) were randomly assigned to the whole plot within each block, and temperature treatments (control/CT and heat/HT) were assigned to the subplots within each whole plot. Two seeds were sown in separate pots filled with Rothamsted Standard compost (75% medium grade peat; 12% screened sterilized loam; 3% medium grade vermiculite; 10% 5 mm screened lime-free grit) and fertilized with Osmocote Exact for 3–4 months at the rate of 3.5 kg/m^3^. One week after sowing, seedlings were thinned down to one per pot and grown under natural light glasshouse conditions with a 16-h light period; they were supplemented with artificial light (230W LED; Kroptek Ltd., London, United Kingdom) if natural light intensity fell below 175 μmol/m^2^/s^1^. The temperature in the glasshouse was set at 21°C/15°C (day/night, actual value: 21.5 ± 0.4/16.3 ± 0.5°C) and the relative humidity (RH) was around 60/75% (day/night) (Supplementary Figure 1A). At the booting stage, plants with the primary tiller reached the targeted AIL (Supplementary Figure 2A) of 6 cm (actual value for each genotype in Supplementary Figure 2B) and were sequentially moved into Fitotron Modular Plant Growth Chambers (HGC1514; Weiss Technik UK Ltd., Loughborough, United Kingdom) for HT treatment (36.97 ± 0.03/26.95 ± 0.17°C, day/night). The light period was 16 h and the intensity was maintained around 600 μmol/m^2^/s^1^ at the plant level. The RH was maintained between 70 and 75% (Supplementary Figure 1D). Plants for CT treatment were kept in the glasshouse. After 5 days of HT treatment, heat-stressed plants were moved back to the glasshouse until final harvest.

#### Exp2

This experiment was conducted between August and December of 2020 and with the same set of 14 genotypes (Supplementary Table 1). The experiment design and plant cultivation conditions were similar as in Exp1. The temperature in the glasshouse was also set at 21°C/15°C (day/night, actual value: 20.6 ± 0.8/15.4 ± 0.7°C) and the RH was around 57/69% (day/night) (Supplementary Figure 1B). When the AIL of the primary tiller reached 2–3 cm (actual value for each genotype in Supplementary Figure 2C), plants for HT treatment were sequentially moved into the same growth chamber as Exp1 with the temperature of 37.02 ± 0.01/27.00 ± 0.01°C (day/night) (Supplementary Figure 1E). Plants for CT treatment were also moved into a similar growth chamber with the temperature of 21.01 ± 0.01/15.01 ± 0.01°C (Supplementary Figure 1E). The light period, intensity, and RH were similar between CT and HT treatments with settings of 16 h, 600 μmol/m^2^/s^1^, and 70–75% respectively. After 5 days in the growth chambers, plants were moved back to the glasshouse until final harvest.

#### Exp3

This experiment was conducted between January and May of 2021 and with 22 genotypes (Supplementary Table 1). The experiment design and plant cultivation conditions were the same as the previous two experiments. The temperature in the glasshouse was also set at 21°C/15°C (day/night, actual value: 21.7 ± 0.4/15.4 ± 0.3°C) and the RH was around 42/49% (day/night) (Supplementary Figure 1C). When the AIL of the primary tiller reached 2–3 cm (actual value in Supplementary Figure 2D), plants for HT treatment were sequentially moved into the growth chamber with a mild temperature of 34.00 ± 0.02/28.00 ± 0.01°C (day/night) (Supplementary Figure 1F). Plants for CT treatment were also moved into a similar growth chamber with the temperature of 21.01 ± 0.02/15.01 ± 0.01°C (Supplementary Figure 1F). The light period, intensity, and RH were similar between CT and HT treatments with settings of 16 h, 600 μmol/m/s, and 70–75% respectively. After 5 days in the growth chambers, plants were moved back to the glasshouse until final harvest.

### Morphological, Phenological, and Physiological Measurements

On the day before (day 0) and after (day 6) HT treatment, the primary tiller of each plant was tagged and measured for AIL. Plant height (PH) was also recorded at these two time points in Exp2 and Exp3 (Supplementary Figure 1G). Chlorophyll SPAD and Fv/Fm (maximum potential quantum efficiency of Photosystem II) were measured at the same time as AIL and weekly thereafter (Supplementary Figure 1G). The SPAD measurement was performed with an MC-100 Chlorophyll Concentration Meter (Apogee Instruments, Inc., Logan, UT, United States). Fv/Fm was measured with a Pocket PEA (Hansatech Instruments Ltd., Norfolk, United Kingdom) after 15–20 min dark adaptation. For each plant, the mean SPAD value of measurements at the tip, middle, and bottom of flag leaf was obtained and one measurement of Fv/Fm was made in the middle of flag leaf. After the HT treatment, the date of each plant was recorded to calculate days to heading in Exp2 and Exp3. Physiological maturity of the spike on the tagged tiller was recorded as days to maturation. These measurements were conducted with four biological replicates of each genotype and treatment combination.

### Measurement With Tagged Tillers/Spikes for Pollen Fertility and the Number of Grains Per Spike

During anthesis in Exp2 and Exp3, the fourth or fifth spikelet (counted from the bottom) was sampled from the tagged tiller. One anther from two florets at the bottom was photographed for a representative image and length of the anther was measured. In Exp3, the remaining five anthers from two florets at the bottom were pooled together for pollen viability analysis using staining with Lugol’s solution. Fully stained pollen was scored as viable, whereas partially stained or aberrant shaped pollen was scored as non-viable. At maturity, the number of filled grains of the tagged spike was counted and recorded as the number of grains per spike, and also, spike length (cm) and number of spikelets were measured. Four biological replicates were used for these measurements.

### Measurement of Tillering Dynamics

In Exp1, development of extra young spikes after heat stress was observed. In Exp2 and Exp3, tiller number was therefore continuously counted for four biological replicates of CT and HT-treated plants of each genotype on the day (day 0) before and after (day 6) the 5-day HT treatment, and on weekly intervals thereafter until maximum tillering (Supplementary Figure 1G).

### Yield-Related Measurements at Maturation

At maturity, the spikes per plant were distinguished into “old” spikes (labeled just before starting the HT treatment in Exp2 and Exp3) and “new” spikes, harvested separately, and then dried in oven at 40°C for 7 days prior to mechanical threshing and cleaning. The weight, number, length, and width were then determined for grain samples from old and new spikes separately with a scale and a MARViN digital seed analyzer (MARViTECH GmbH., Wittenburg, Germany). Grain yield per plant was calculated as the sum of grains from old and new spikes. The aboveground biomass for each plant was determined as the weight of all straw materials dried in an oven at 80°C for 48 h. The yield-related measurements were analyzed with four biological replicates.

### Statistical Analysis

The data from the time course SPAD measurements were fitted using a generalized additive model (GAM) for each of the three experiments to estimate maximum SPAD (SPADmax), senescence onset (SenOnset), and senescence rate (SenRate) (Supplementary Figure 3). SPAD was predicted by a smooth function of time (days counted from stress initiation), with a separate smooth function fitted for each combination of genotype and treatment. The Exp1 model used eight basis functions, whereas Exp2 and Exp3 used seven basis functions. SPADmax was estimated from the fitted predicted model. SenOnset was calculated as the day that SPAD fell to 95% of the maximum SPAD. Senescence period was defined over 14 days from the onset or until the end of the measurement, whichever was shorter. SenRate was then calculated as the daily reduction of SPAD over the senescence period. GAMs were fitted in R package (version 3.6.1) using the “mgcv” package (version 1.8-35) (Wood, 2011).

All trait measurements and calculated parameters (Supplementary Table 2) were used for statistical analysis in R 4.0.3.^1^. First, descriptive statistics were summarized with the “describeBy” from the “psych” package (Revelle, 2020). The effects of genotype treatment and the interaction were obtained from ANOVA with the model fitted with “lmer” from the R package “lmerTest” (Kuznetsova et al., 2017); genotype, treatment, and their interaction were treated as fixed factors, while block and genotype nested in block were treated as random effects. Later, Tukey’s *post hoc* test was carried out for multiple test comparisons to identify genotypic variation. Estimated marginal means were calculated for each combination of genotype and treatment. Subsequently, for either CT or HT treatment, Pearson correlation coefficient table was calculated by using “tab_corr” from “sjPlot” package (Lüdecke, 2021) among measurements and pairwise-deletion method was used to account for missing data. For each experiment and temperature treatment, correlations among different traits were visualized as networks with the “qgraph” package (Epskamp et al., 2012).

## Results

### Heat-Impaired Pollen Fertility and Number of Grains Per Spike

**FIGURE 1:**
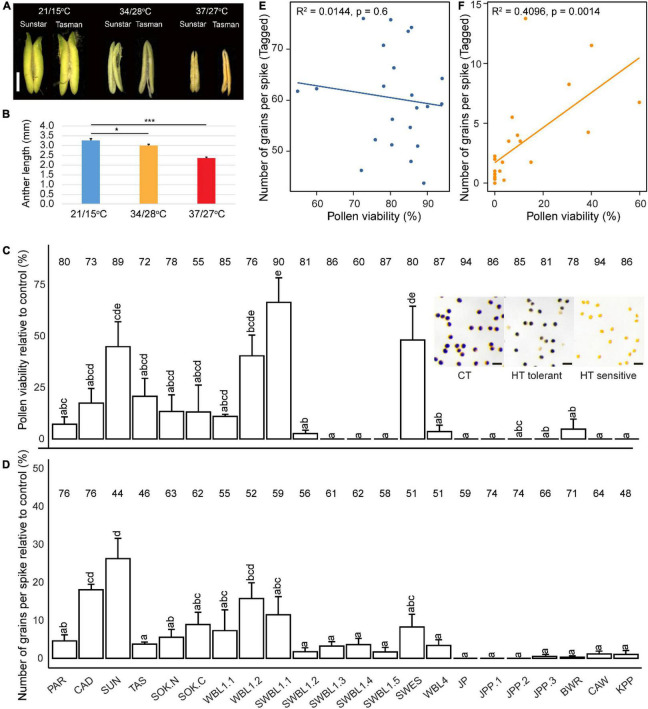
The heat effects on wheat reproductive traits. Anther morphology after heat treatments in two representative genotypes is shown in (**A**, bar = 1 mm). Average anther length across all analyzed genotypes in Exp2 (37/27°C) and Exp3 (34/28°C) is shown in **(B)**. Genotypic variation of pollen viability **(C)** and the number of grains per spike **(D)** under heat in Exp3. Representative pollen images for CT, HT-tolerant, and HT-sensitive were inserted (**C**, bar = 100 μm). The data were obtained from the primary tiller and calculated as relative to control values, which are shown on the top of each bar. Pearson correlation between pollen viability and number of grains per spike under control **(E)** and heat **(F)**. Significance level: ^***^p < 0.001; ^**^p < 0.01; *p < 0.05; *p < 0.05.

To understand the effects of heat on pollen development and grain setting, the primary tiller of each plant was tagged and measured. In Exp1 and Exp2, the imposed severe heat treatment of 37°C/27°C caused nearly complete loss of grain setting for all genotypes, except for Paragon and Cadenza (Supplementary Figure 4). The anther morphology was also severely changed by the HT treatments indicating complete absence of viable pollen (Figures 1A,B). In Exp3, relatively mild heat stress (34°C/28°C) also significantly reduced anther length; however, this was less severe compared with Exp2 (Figures 1A,B) and pollen viability was therefore analyzed by staining with Lugol’s solution. The results showed considerable variation among genotypes, ranging from 0 to 60%. One line (SWBL1.1, a progeny between the cross of Sokoll and Weebill1) had the highest pollen viability (relative to control value), followed by SWES, SUN (Sunstar), and WBL1.2 (Weebill1) (Figure 1C). The number of grains of the tagged primary spike was also variable among the genotypes, with SUN showing the highest value relative to control value (Figure 1D). Further analysis found a positive correlation between pollen viability and the number of grains per spike under HT (Figure 1F), but not under CT treatment (Figure 1E).

### Heat-Stimulated Tillering/Spike Formation and Its Association With Yield

**FIGURE 2:**
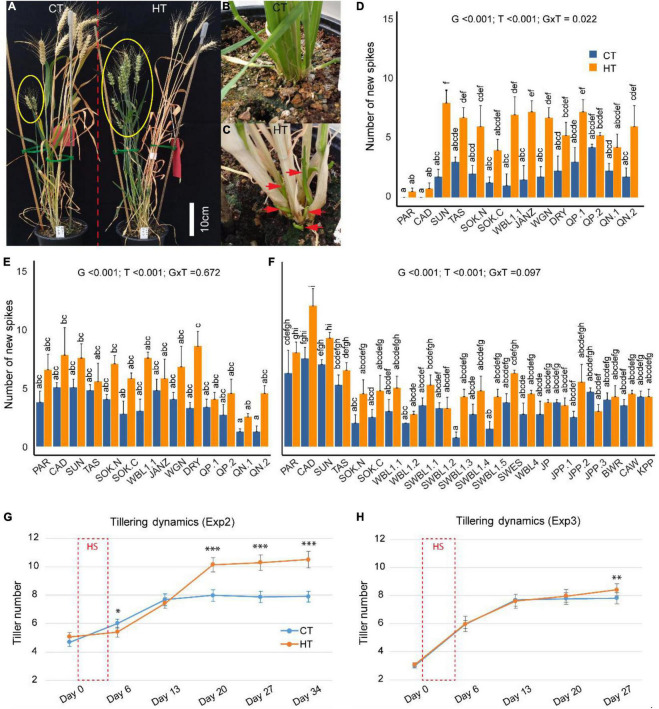
The effects of heat treatments on tillering/spike formation. After heat (HT) treatment, new tillers/spikes (in yellow ellipse) were vigorously stimulated, while controls were not (CT, **A**). Tiller outgrowth from CT **(B)** and HT **(C)** around 1 week after stress treatment. A comparison of the number of new spikes between CT and HT in Exp1 **(D)**, Exp2 **(E)**, and Exp3 **(F)**. The effects of genotype/G, treatment/T, interaction/G × T were indicated for each panel. Dynamic change of tillering before (day 0), after (day 6) the 5-day HT treatment (days 1–5), and weekly intervals in Exp2 **(G)** and Exp3 **(H)**. Significance level: ***p < 0.001; **p < 0.01.

During the ripening stage of Exp1, the senescence status of tillers/spikes was clearly separated into two groups (Figure 2A) and tillers were therefore distinguished into old (pre-heat) and new (post-heat) spikes for each plant. About 1 week after heat treatment, new tiller outgrowth was noticed from the bottom of HT-stressed plants (Figures 2B,C). A final count of spikes found significantly more new spikes in the HT-treated plants compared with the CT plants in Exp1 (*p* < 0.001), Exp2 (*p* < 0.001), and Exp3 (*p* < 0.001) (Figures 2D–F), while the number of old spikes was similar between HT and CT conditions (Supplementary Figure 5). In addition, there was no significant interaction between treatment and genotype (Figures 2D–F), indicating that all genotypes responded similarly to the HT treatment in terms of new spike formation. The analysis of tillering dynamics in Exp2 and Exp3 showed that onset of new tiller development commenced at 2–3 weeks after the HT treatment, with a stronger effect observed in Exp2 (Figures 2G,H). Moreover, the more severe heat stress in Exp2 (37°C/27°C) also caused tiller retardation on day 6, 1 day after the end of the HT treatment (Figure 2G), but this was not observed under the milder heat stress condition in Exp3 (34°C/28°C) (Figure 2H).

**FIGURE 3:**
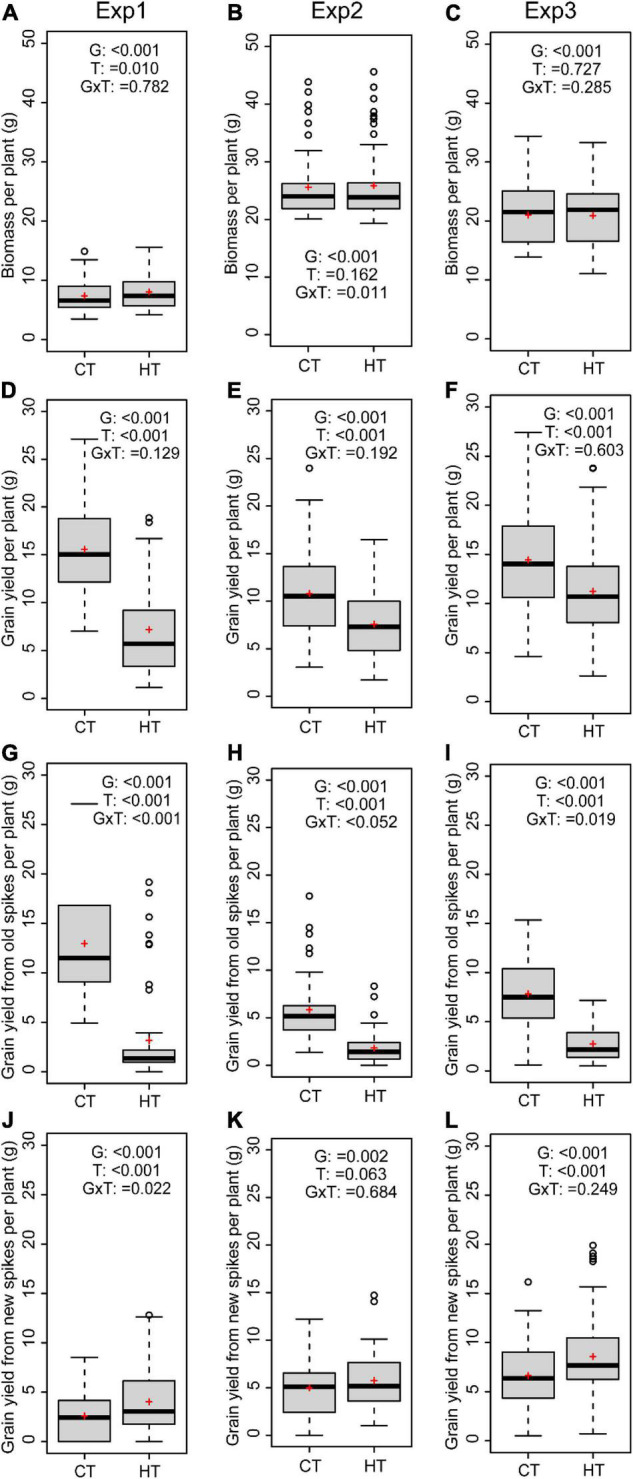
Average values of wheat genotypes grown under high temperature (HT) or control (CT) across the three experiments are shown for biomass **(A–C)**, grain yield per plant **(D–F)**, grain yield from old spikes **(G–I)**, and grain yield from new spikes **(J–L)**. The effects of genotype (G), treatment (T), and interaction (G × T) are indicated for each panel.

As new tillers developed after the HT treatment and extended the days to maturity of the plants, the aboveground biomass per plant (including both old and new tillers) was very similar between HT and CT treatments (Figures 3A–C). Nevertheless, the overall grain yield per plant was significantly reduced after the HT treatment in all three experiments (*p* < 0.001 for all) (Figures 3D–F). This was primarily due to heat-induced sterility in the old spikes (Figures 3G–I). However, heat-induced formation of new spikes gave rise to similar (Exp2, Figure 3K) or even significantly higher grain yield from new spikes in Exp1 and Exp3 (Figures 3J,L). The proportion of yield from new spikes after the HT treatment was therefore significantly higher than under CT conditions (Supplementary Figure 6). As sink size was reduced by limited seed setting of old spikes under HT condition, source supply became more than sufficient for the survived developing grains, and their width and length were significantly higher than grains of old spikes from control plants (Supplementary Table 2). By contrast, the grains from new spikes showed variable responses in terms of width and length (Supplementary Table 2).

### Heat Effects on Plant Morphology, Phenology, and Chlorophyll Dynamics

**FIGURE 4:**
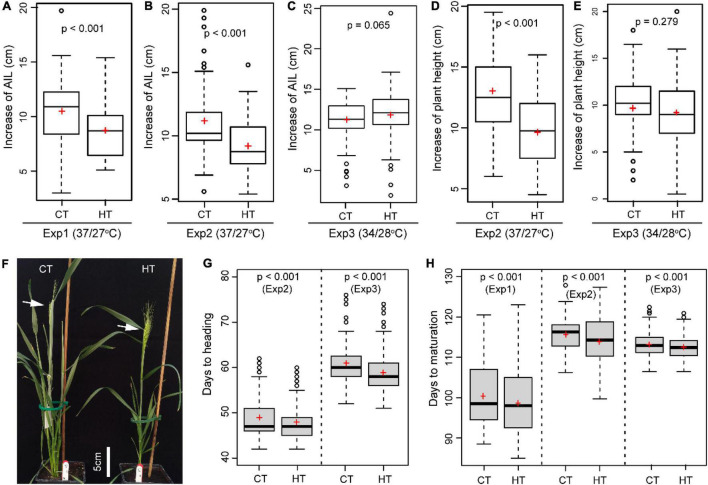
The heat effects on plant morphology and phenology. Comparison of the effect of heat (HT) and control (CT) treatments on the increase of auricle interval length (AIL, **A–C**), the increase in plant height **(D,E)**, days to heading **(F,G)**, and days to maturation **(H)**.

When wheat plants were exposed to heat stress during the early booting stage, the increase in AIL (*p* < 0.001 for Exp1 and Exp2) and PH (*p* < 0.001 Exp2) during the 5-day treatments was significantly reduced by the HT of 37°C/27°C

compared with the CT of 21°C/15°C (Figures 4A,B,D). In contrast, the AIL (*p* = 0.065) and PH (*p* = 0.279) were marginally affected under the milder HT of 34°C/28°C in Exp3 (Figures 4C,E). HT treatments also changed plant phenology as indicated by the significantly reduced number of days to heading (DTH) (*p* < 0.001 for Exp2 and Exp3) (Figures 4F,G) and days to maturation (DTM) (*p* < 0.001 for Exp1, Exp2, and Exp3) (Figure 4H).

**FIGURE 5:**
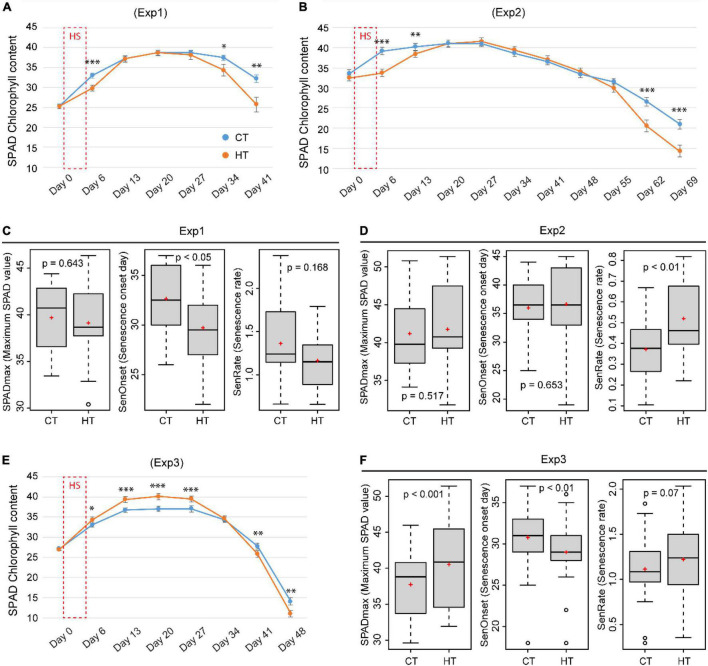
The effects of heat on SPAD (chlorophyll content index) and leaf senescence parameters. Comparison of the effect of heat (HT) and control (CT) treatments on the dynamic change of SPAD measured before (day 0) and 1 day after (day 6) a 5-day HT treatment, and in weekly intervals thereafter in Exp1 **(A)**, Exp2 **(B)**, and Exp3 **(E)**. Senescence-related parameters, SPADmax (maximum SPAD value), SenOnset (Senescence onset day), and SenRate (Senescence rate), were compared between CT and HT for Exp 1 **(C)**, Exp2 **(D)**, and Exp3 **(F)**. Significance level: ^***^p < 0.001; ^**^p < 0.01; *p < 0.05.

To understand the physiological basis of changes in phenology, dynamic changes in SPAD and Fv/Fm were compared between CT and HT treatments. On day 6 (1 day after treatment), in comparison to the corresponding CT conditions, SPAD value was significantly reduced by the severe heat (37°C/27°C) in Exp1 (Figure 5A) and Exp2 (Figure 5B), but surprisingly increased slightly after the mild heat (34°C/28°C) in Exp3 and maintained a higher maximum SPAD value (Figures 5E,F). At later stages, however, an accelerated decrease in SPAD was observed under HT conditions in all three experiments, irrespective of heat stress intensity (Figures 5A,B,E). Based on the time course of SPAD measurements, GAMs were fitted to estimate maximum SPAD (SPADmax), senescence onset (SenOnset), and senescence rate (SenRate) for each combination of genotype and treatment. In Exp1 and Exp3, SenOnset from HT treatment was reproducibly and significantly advanced in comparison with CT conditions, whereas SenRate was similar between treatments (Figures 5C,F). By contrast, Exp2 showed an opposite response with similar SenOnset between treatments, but an increased SenRate under HT (Figure 5D). This variation between the three experiments may be due to variable intensities of natural sunlight. Even within the same experiment, some genotypes showed earlier SenOnset, while others showed faster SenRate under HT treatment (Supplementary Figures 7–9). In addition, the Fv/Fm value at day 6 was always significantly reduced by HT treatment in all three experiments indicating a negative effect of the HT on PSII (Supplementary Figure 10).

### Analysis of Trait Correlations From Different Experiments and Temperature Conditions

**FIGURE 6:**
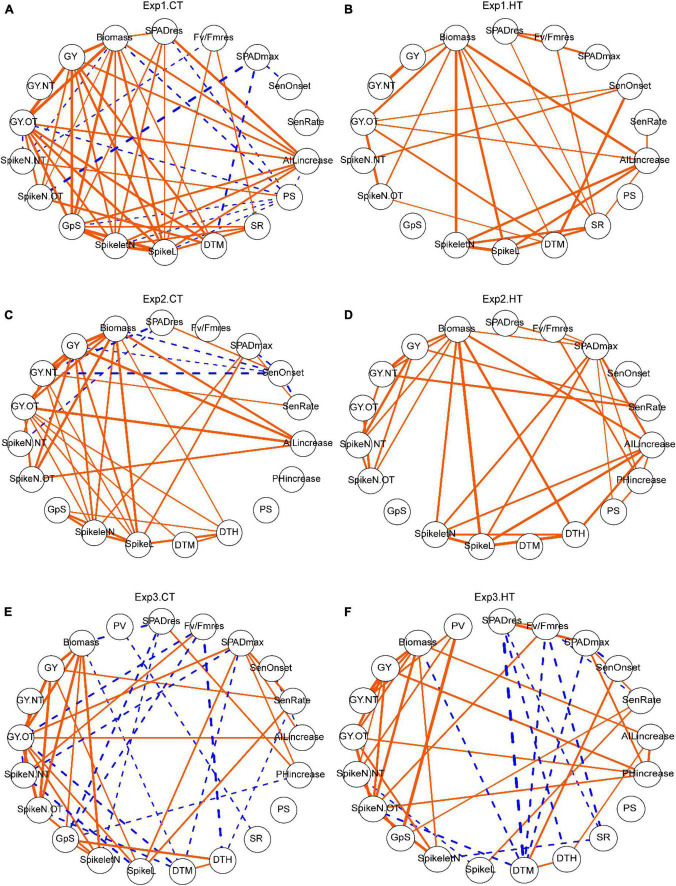
The effects of heat on trait relationships across different experiments. Correlation networks for Exp1 **(A,B)**, Exp2 **(C,D)**, and Exp3 **(E,F)**. Only significant correlations are shown and the width of the edges indicated correlation *r*-value. Orange solid edges represent positive correlations, while blue dashed edges represent negative correlations. Trait abbreviations: *SPADres*: (SPAD at day 6—SPAD at day 0)/SPAD at day 0; *Fv/Fmres*: (Fv/Fm at day 6 -Fv/Fm at day 0)/(Fv/Fm at day 0); *SPADmax*, maximum value of SPAD; *SenOnset*, senescence onset time; *SenRate*, senescence rate; *AILincrease*, auricle interval length increase during the 5-day treatment; *PHincrease*, plant height increase during the 5-day treatment; *PS*, observed frequency of paired spikelet from all spikes; *SR*, observed frequency of sham ramification from all spikes; *DTH*, days to heading; *DTM*, days to maturation; *SpikeL*, spike length (tagged primary spike); *SpikeletN*, number of spikelets (tagged primary spike); *GpS*, number of grains per spike (tagged primary spike); *SpikeN.OT*, number of spikes from old tillers; *SpikeN.NT*, number of spikes from new tillers; *GY.OT*, grain yield of old tillers; *GY.NT*, grain yield of new tillers; *GY*, grain yield per plant (sum of old and new tillers); *Biomass*, the dry weight of all straw per plant; *PV*, pollen viability from the middle spikelet of tagged spike.

To understand the relationships among different traits across genotypes, correlations were calculated (Supplementary Table 3) and visualized as networks (Figure 6). Number of grain per spike (GpS) showed different correlations under control and HT conditions; In Exp1 and Exp2, there was no correlation between GpS and any other trait under HT, but under CT, it was strongly and positively correlated with the number of spikelets (SpikeletN) (*r* = 0.98^***^ for Exp1 and 0.81^**^ for Exp2) and length (SpikeL) (*r* = 0.94^***^ for Exp1 and 0.79^**^ for Exp2) of the tagged spike, as well as with biomass (*r* = 0.88^***^) and yield (*r* = 0.88^***^) in Exp1. In Exp3, GpS was also associated with different traits between CT and HT. The importance of induced new tillers and spikes after heat stress was corroborated by the reproducible strong positive correlations (*r* = 0.98^***^, 0.92^***^, 0.80^***^ for Exp1, Exp2, and Exp3, respectively) between grain yield of new tillers (GY.NT) and total grain yield (GY), observed in all three experiments (Figure 6 and Supplementary Table 3). This suggests a critical role of new spikes in mitigating heat-induced yield reduction. In addition, the morphological traits, increase in AIL and PH, were generally positively correlated with yield or biomass-related traits, regardless of temperature treatments. Ultimately, SPAD and Fv/Fm parameters were not consistently correlated with other traits from different experiments and treatments. In Exp1, SenOnset showed HT-specific weak positive correlations with a grain yield of old tillers (GY.OT) (*r* = 0.62*) and a spike number of new tillers (SpikeN.NT) (*r* = 0.69*); SenRate was closely related to yield traits in both Exp2 (*r* = 0.62* with GY.NT under CT; *r* = 0.79^**^ with GY.NT and 0.69* with GY under HT) and Exp3 (*r* = 0.47* with GY and 0.53* with SpikeL under CT; *r* = 0.45* with GpS and 0.49* with SpikeL under HT), but not heat-specific; in Exp2, SPADmax was important, as it was strongly correlated with Spikelet (*r* = 0.66* under CT, 0.75^**^ under HT) and SpikeL (*r* = 0.70* under CT, 0.74^**^ under HT) of tagged spike (Figure 6 and Supplementary Table 3).

## Discussion

### Importance and Limitation of Pollen Viability as a Target Trait for Wheat Heat Research

In the present study, anther morphology was gradually affected under two levels of heat stress, 34°C/28°C (day/night) and 37°C/27°C, applied for 5 days during early booting stage coinciding with pollen development. The more severe heat stress condition in this study led to a complete loss of pollen viability, while results from a parallel study, in which the same 37°C/27°C heat treatment was applied that lasted for only 3 days (Erena, 2018), were similar to the 5-day, milder temperature (34°C/28°C) treatment, suggesting that both stress intensity and duration are critical to screening reproductive heat tolerance. Under the 34°C/28°C condition, pollen viability was considerably variable among genotypes. Two of the lines (SWB1.1 and SWES) with high pollen viability share one common parent, Sokoll, in their pedigree. Sokoll is an advanced wheat line derived from synthetic hexaploid wheat and has shown a yield advantage under terminal heat stress in other reports (Cossani and Reynolds, 2015; Thistlethwaite et al., 2020), although it did not show particularly high pollen viability after early booting-stage heat stress in this study. These results suggest stage-specific heat tolerance; therefore, it is necessary to pyramid tolerant traits across different developmental stages. Another parental line included in this study, Weebill1 (WBL1.1 and WBL1.2), has previously been reported to be tolerant to a wide range of variable environmental conditions (Singh et al., 2007). One of the most tolerant genotypes identified in this study was Sunstar, in agreement with data reported by Erena (2018) who also demonstrated the reproductive heat tolerance of Sunstar. These identified genotypes with heat tolerance during pollen development may be suitable donors for breeding and warrant further studies to understand the underlying genetic and molecular-physiological mechanisms. The importance of pollen viability is supported by its positive correlation with the number of grains per spike under heat stress. Interestingly, similar relationships have been reported in other crops (Xu et al., 2017; Shi et al., 2018) and abiotic stresses (Ji et al., 2010), indicating that pollen fertility is a general limiting factor for final grain number under suboptimal growth conditions. Therefore, it should be an important target trait for heat-related research and breeding. Nevertheless, the response of pollen viability to heat stress is highly dependent on the developmental stage when stress is applied (Saini and Aspinall, 1982; Prasad and Djanaguiraman, 2014) and it is thus important to consider genotypic differences and carefully target meiosis to microspore stage when applying heat stress to exclude confounding effects. Currently, the most widely used morphological marker for pollen developmental stage is AIL, which is also known as auricle distance (Ji et al., 2010; Erena, 2018; Bokshi et al., 2021). However, AIL corresponding to a specific pollen developmental stage varies among different genotypes (Erena, 2018) and must be determined for each genotype, which is laborious. Fortunately, progress has been made by non-destructive X-ray micro-computed tomography scanning (Fernández-Gómez et al., 2020), and integrating this with modeling could be a promising way to overcome difficulties with accurate identification of developmental stages of wheat pollen.

### Utilizing Developmental Plasticity to Mitigate Heat Effects on Yield

The number of spikes per plant, interacting with spikelet number and floret fertility, determines grain number and thereby final yield. Our data show that a short episode of heat stress during early booting stage induced the development of new tillers and spikes, which is in agreement with other studies (Bányai et al., 2014; Chavan et al., 2019; Hütsch et al., 2019). Although tillering was initially inhibited under severe heat stress, new tillers started emerging at 2 weeks after recovery, corresponding to about 1 week after anthesis. This timing suggests that available photosynthates stored in vegetative tissue that cannot be translocated into grain due to spikelet sterility can be reallocated into the development of new tillers and spikes. Additional photo-assimilates for new tillers and spikes would be produced during recovery and this is reflected by its positive correlation with delayed onset of senescence (Figure 6C). The observed formation of new spikes after heat stress compensating for heat-induced biomass and yield losses under controlled environment conditions now needs to be corroborated under field conditions to ensure that it is a valid target trait for breeding. In addition, a higher frequency of paired spikelets (Boden et al., 2015) and sham ramification (Amagai et al., 2017) was observed in heat-treated plants and this may also be related to excessive source supply. Although these traits were not correlated with yield, they could contribute to understand mechanisms underlying such developmental abnormalities.

### Accelerated Leaf Senescence After Brief Heat Stress During Early Booting Stage

Screening wheat for heat tolerance in the field is generally implemented by late-sowing to impose continuous terminal heat stress during grain filling, often resulting in accelerated leaf senescence (Bergkamp et al., 2018). In the present study, a similar stimulation of flag leaf senescence was observed after a brief episode of heat stress was applied during early booting. It is possible that plants are able to measure and memorize phenology or leaf age to program the senescence process (Woo et al., 2019). In our study, model fitting using SPAD time course data proved to be successful in identifying senescence parameters. Both earlier onset and faster senescence rate were identified and were closely related to accelerated leaf senescence, in agreement with similar results reported by Šebela et al. (2020). Heat-specific positive correlations between senescence onset (SenOnset), new spike formation (SpikeN.NT), and yield of old tillers (GY.OT) in Exp1 support the important role of late senescence. The observed positive associations between senescence rate (SenRate) and yield traits (grain yield/GY, number of grains per spike/GpS, spike length/SpikeL) in Exp2 and Exp3 suggest fast nutrient remobilization in high-yielding lines. Finally, both SPAD and Fv/Fm were reduced by heat immediately after the treatment (day6) in Exp1 and Exp2, but the mild temperature of 34°C/28°C only decreased Fv/Fm, not SPAD. These results indicate that Fv/Fm may be more sensitive and therefore a better parameter for heat tolerance evaluation (Cao et al., 2019). Therefore, these senescence-related parameters are useful for crop phenotyping, and integrating modeling with high-throughput imaging measurements will enable large-scale analysis.

## Conclusion

In this study, a spring wheat panel, including heat-tolerant elite varieties and their pre-breeding lines, was dissected for reproductive, developmental, physiological, and yield responses along with their inter-relationships after a 5-day heat stress application during the early booting stage. In comparison with the control treatment, pollen viability from the tagged primary spike was significantly decreased by heat and subsequently reduced number of grains per spike. The heat stress, however, resulted in late tillering after the disruption of sink strength. Consequently, more new spikes were formed contributing to final yield and biomass, though an additional week was needed for the maturation of the late tillers. Flag leaf SPAD (Chlorophyll content index) and Fv/Fm (maximum potential quantum efficiency of Photosystem II) were reduced by heat stress. Model fitting with time course SPAD measurements showed accelerated leaf senescence by either earlier onset or faster senescence rate, and these parameters were associated with yield traits. Ongoing genomic and genetic studies will subsequently be used to dissect the mechanism of identified heat-tolerant genotypes (Sunstar, SWBL1.1). Taken together, these reproductive, developmental, and physiological traits could be further used as targets for understanding basic mechanisms and breeding heat-tolerant wheat.

## Data Availability Statement

The original contributions presented in the study are included in the article/Supplementary Material, further inquiries can be directed to the corresponding author/s.

## Author Contributions

SH conceived and supervised the project, together with EV-S and MP. JX designed and implemented the experiments and analyzed the data, with support from CL. MR and SD advised on the selection of genotypes included in this study and provided the seeds. SH, EV-S, SH-L, and MP held regular project planning discussions. JX wrote the manuscript, which was reviewed and edited by SH, CL, MR, SD, EV-S, SH-L, and MP. All authors contributed to the article and approved the submitted version.

## Conflict of Interest

The authors declare that the research was conducted in the absence of any commercial or financial relationships that could be construed as a potential conflict of interest.

## Publisher’s Note

All claims expressed in this article are solely those of the authors and do not necessarily represent those of their affiliated organizations, or those of the publisher, the editors and the reviewers. Any product that may be evaluated in this article, or claim that may be made by its manufacturer, is not guaranteed or endorsed by the publisher.

## Footnotes

1
https://www.R-project.org/
