# Supplementary material for: The Effects of Brief Heat During Early Booting on Reproductive, Developmental, and Chlorophyll Physiological Performance in Common Wheat (Triticum aestivum L.)
Source: Front Plant Sci. 2022 May 16;13:886541. doi: 10.3389/fpls.2022.886541 (PMC9149578; doi:10.3389/fpls.2022.886541)
Supplement: Supplementary file 5 [file Data_Sheet_1.docx]

**Supplementary Figures:**


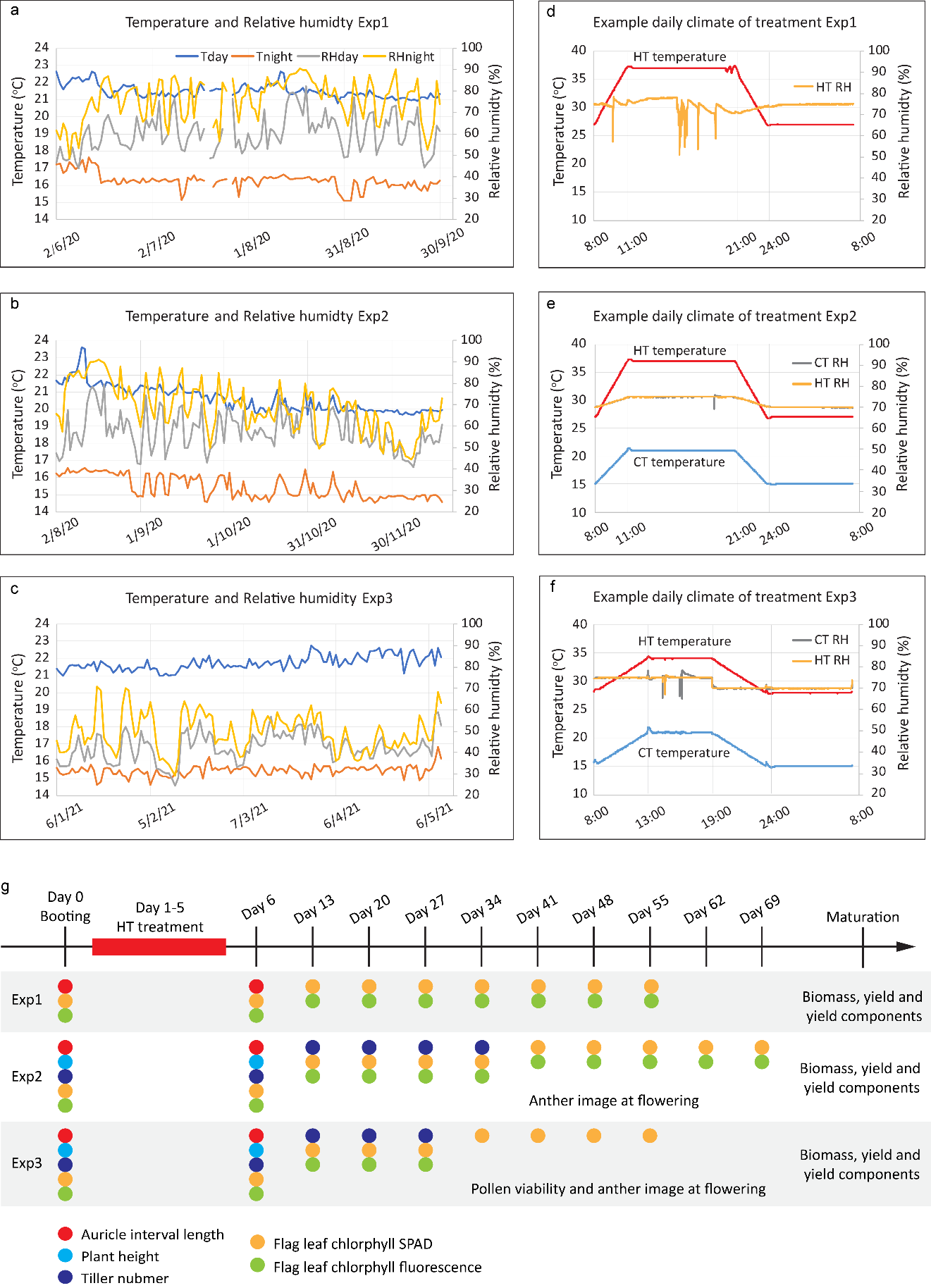


Fig S1. The temperature and relative humidity records during plant growth for Exp1 (a), Exp2 (b) and Exp3 (c). The daily example temperature and relative humidity for control/CT and heat/HT treatment for Exp1 (d), Exp2 (e) and Exp3 (f). The schedule for HT treatment and measurements (g).


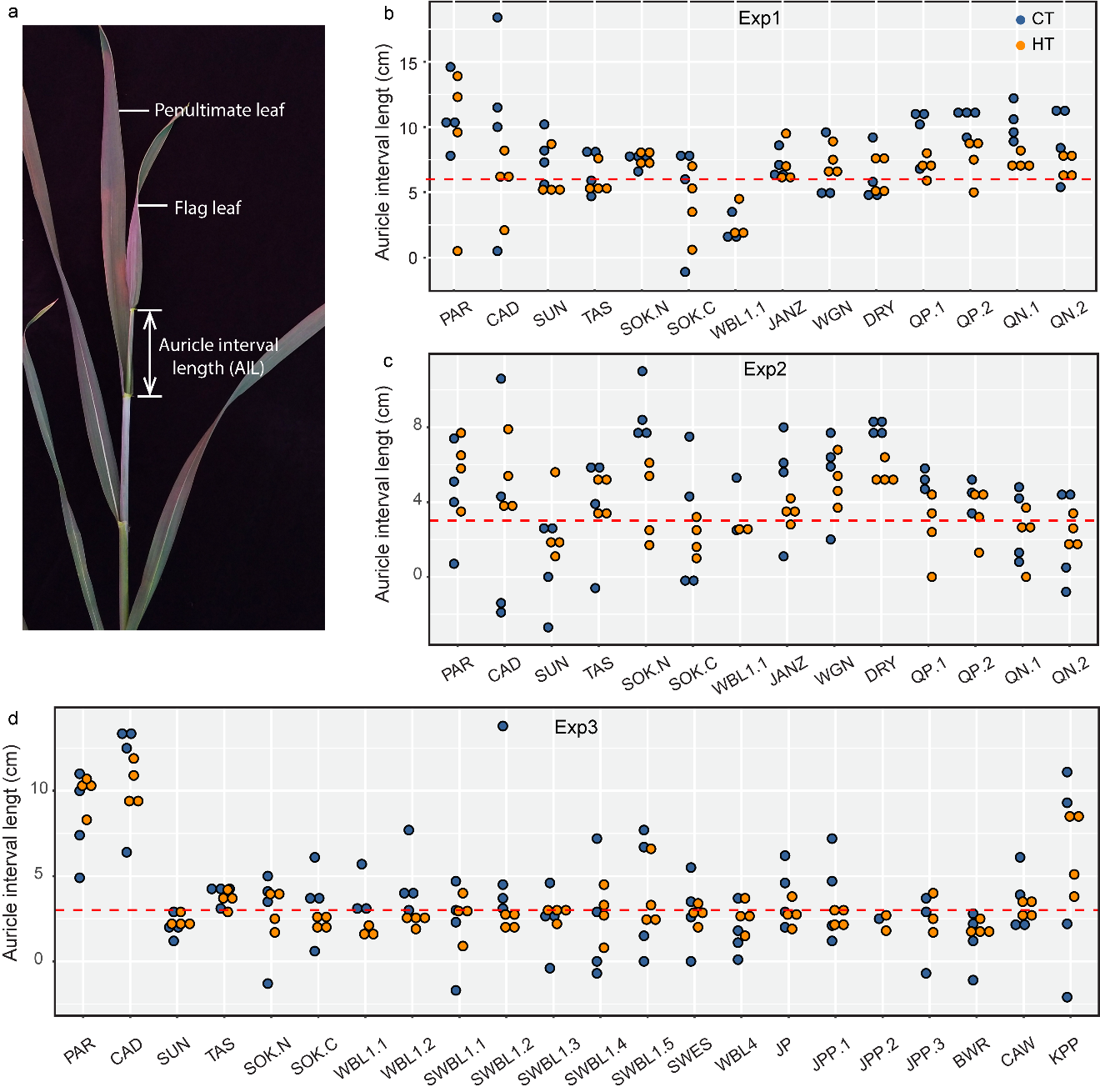


Fig S2. Illustration of auricle interval length (AIL, a), the morphological marker for pollen development. The actual AIL value for each genotype at the initiation of the 5-day heat treatment for Exp1 (b), Exp2 (c) and Exp3 (d).


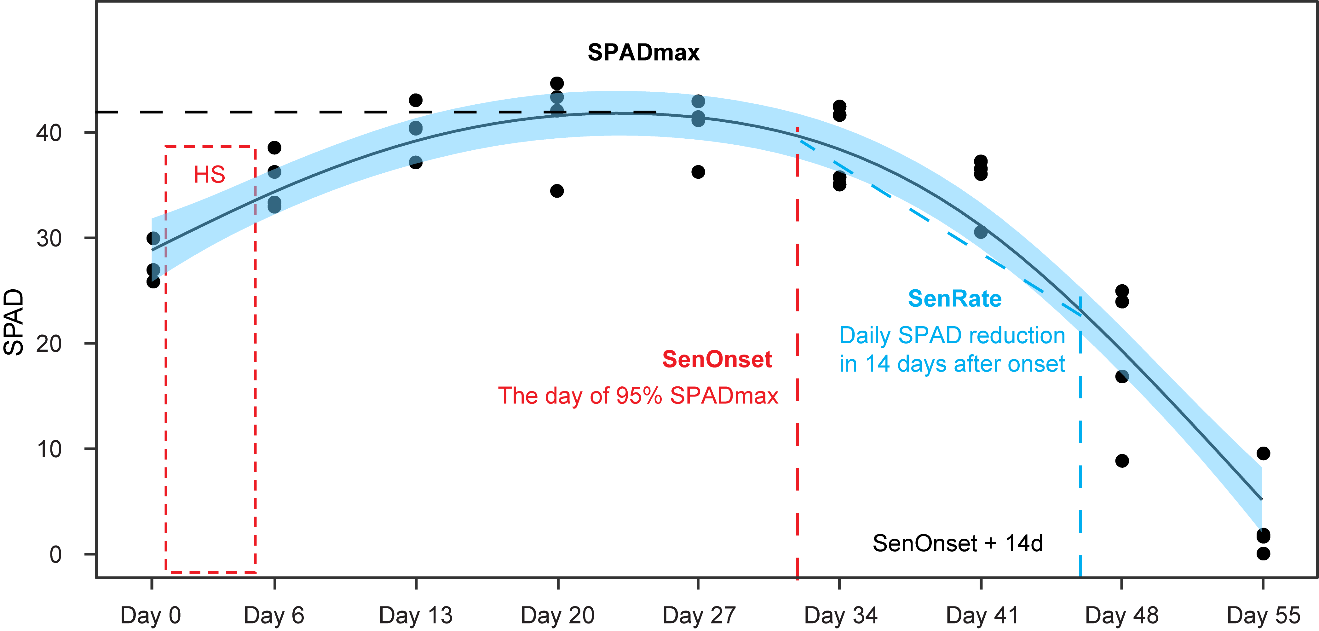


Fig S3. Graphic illustration of parameters predicted from the model fitted with multiple SPAD measurements. SPADmax represents the maximum value of SPAD; the day reaching 95% SPADmax is defined Senescence onset (SenOnset); daily SPAD reduction within 14 days after onset is senescence rate (SenRate).


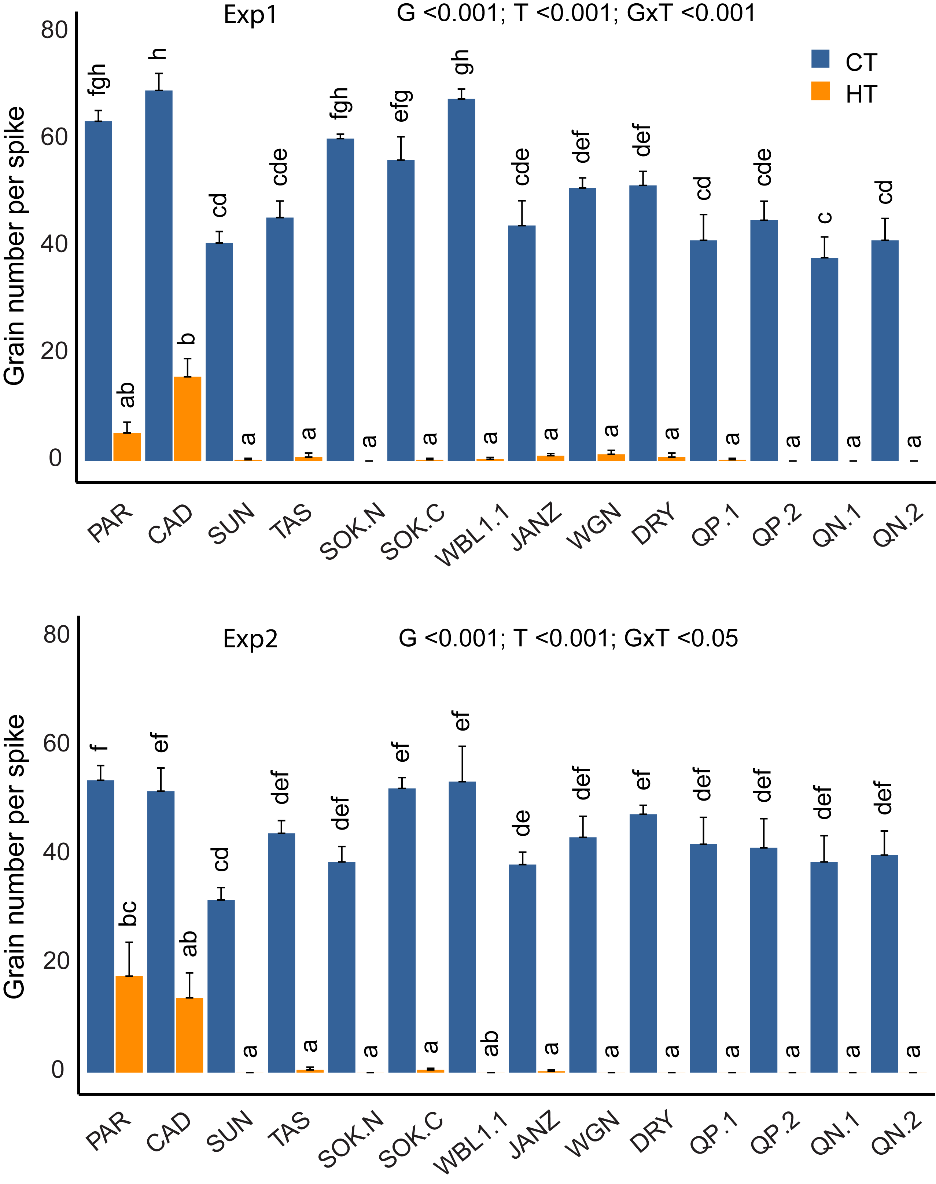


Fig S4. Comparing number of grains per spike between CT and HT in Exp1 and Exp2. The data was obtained from the tagged primary tiller. The effects of genotype/G, treatment/T, interaction/G x T were indicated for each panel.


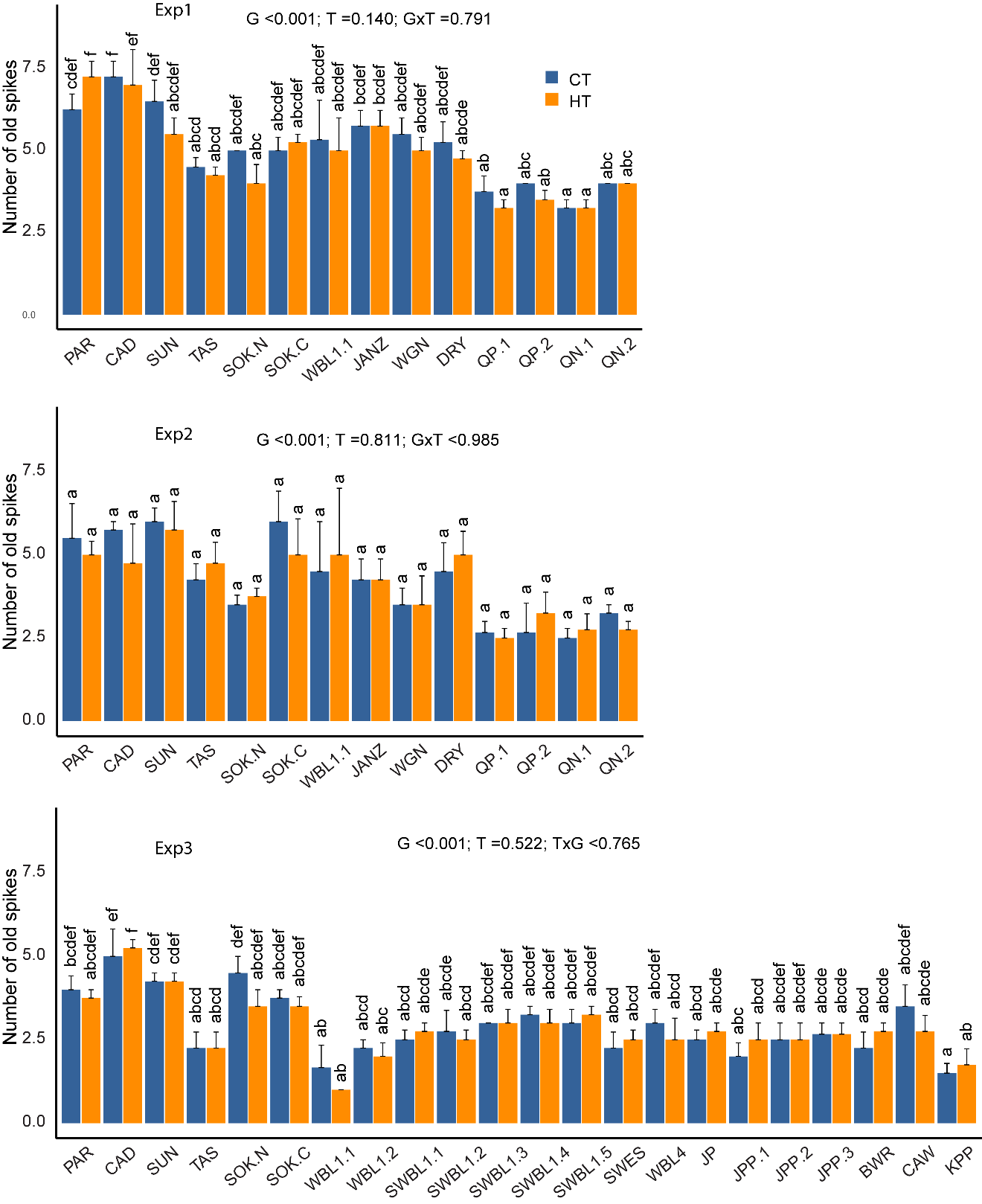


Fig S5. Comparing the number of old spikes between CT and HT in Exp1, Exp2 and Exp3. The effects of genotype/G, treatment/T, interaction/G x T were indicated for each panel.


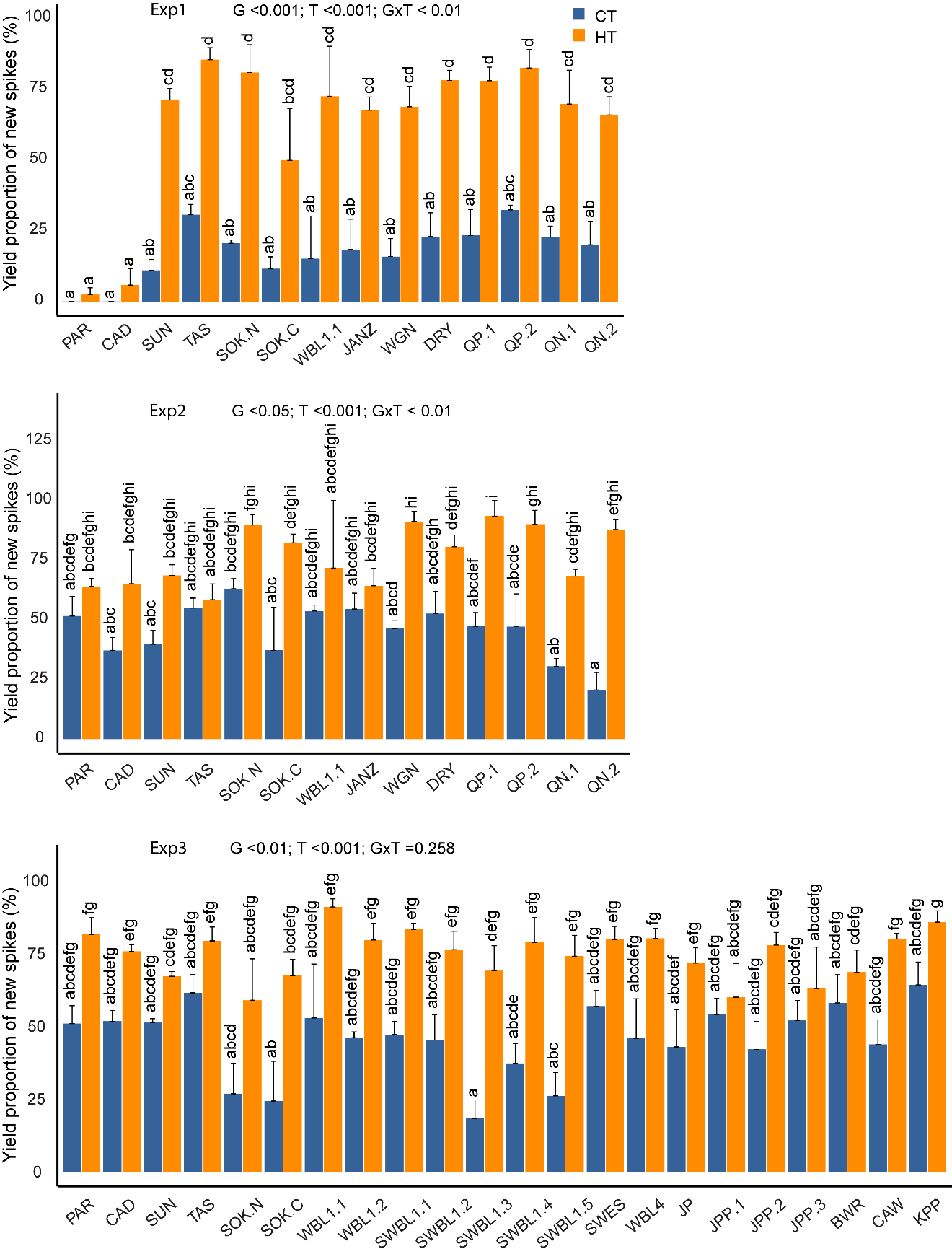


Fig S6. Comparing the yield proportion contributed by new spikes between CT and HT in Exp1, Exp2 and Exp3. The effects of genotype/G, treatment/T, interaction/G x T were indicated for each panel.


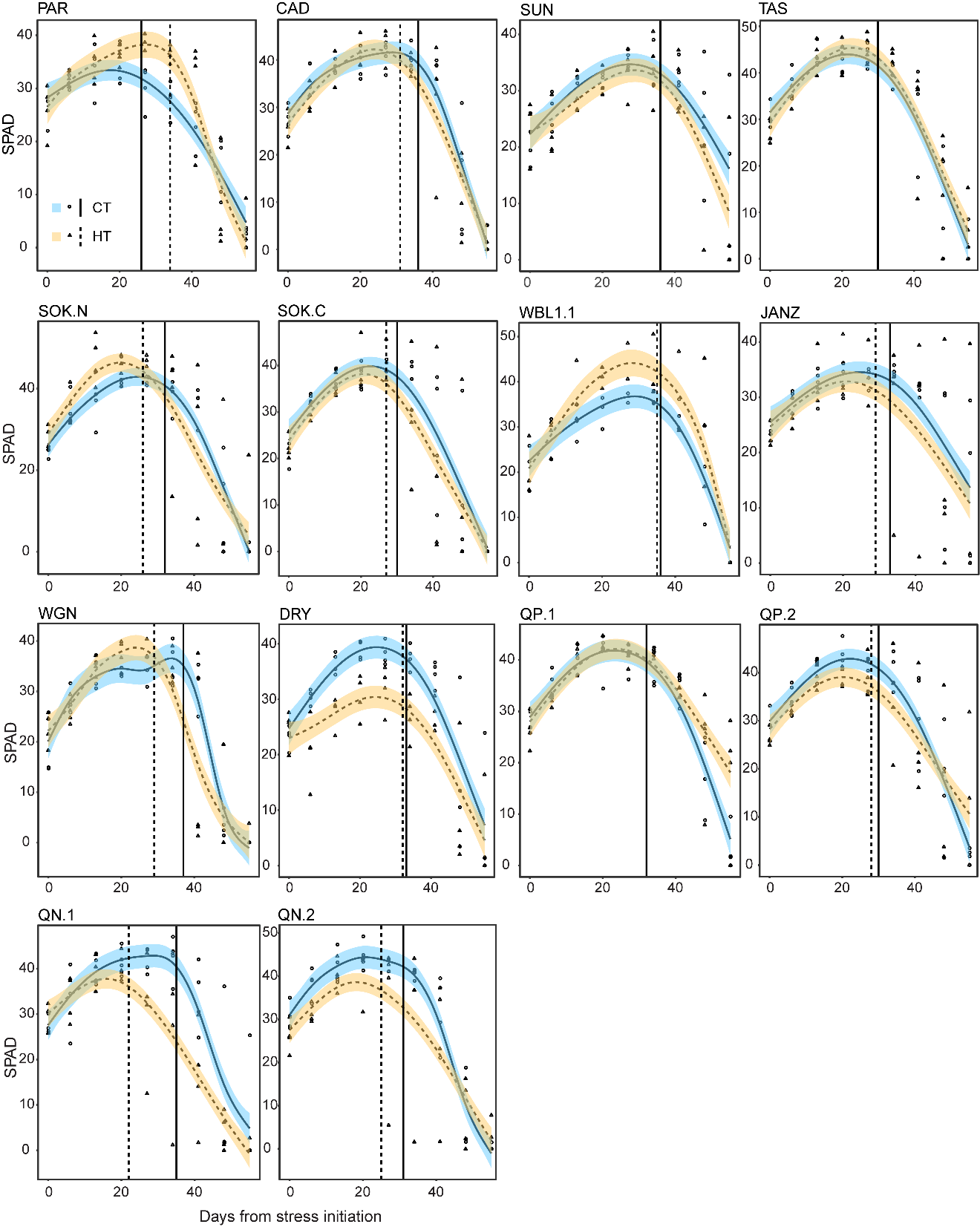


Fig S7. Generalised additive models (GAM) fitting for each genotype in Exp1. The vertical lines (solid, CT; dashed HT) indicate senescence onset (SenOnset). Smooth lines show the mean smooth model estimates, shading around the lines shows 95% confidence of the mean model estimate (blue, CT; yellow, HT).


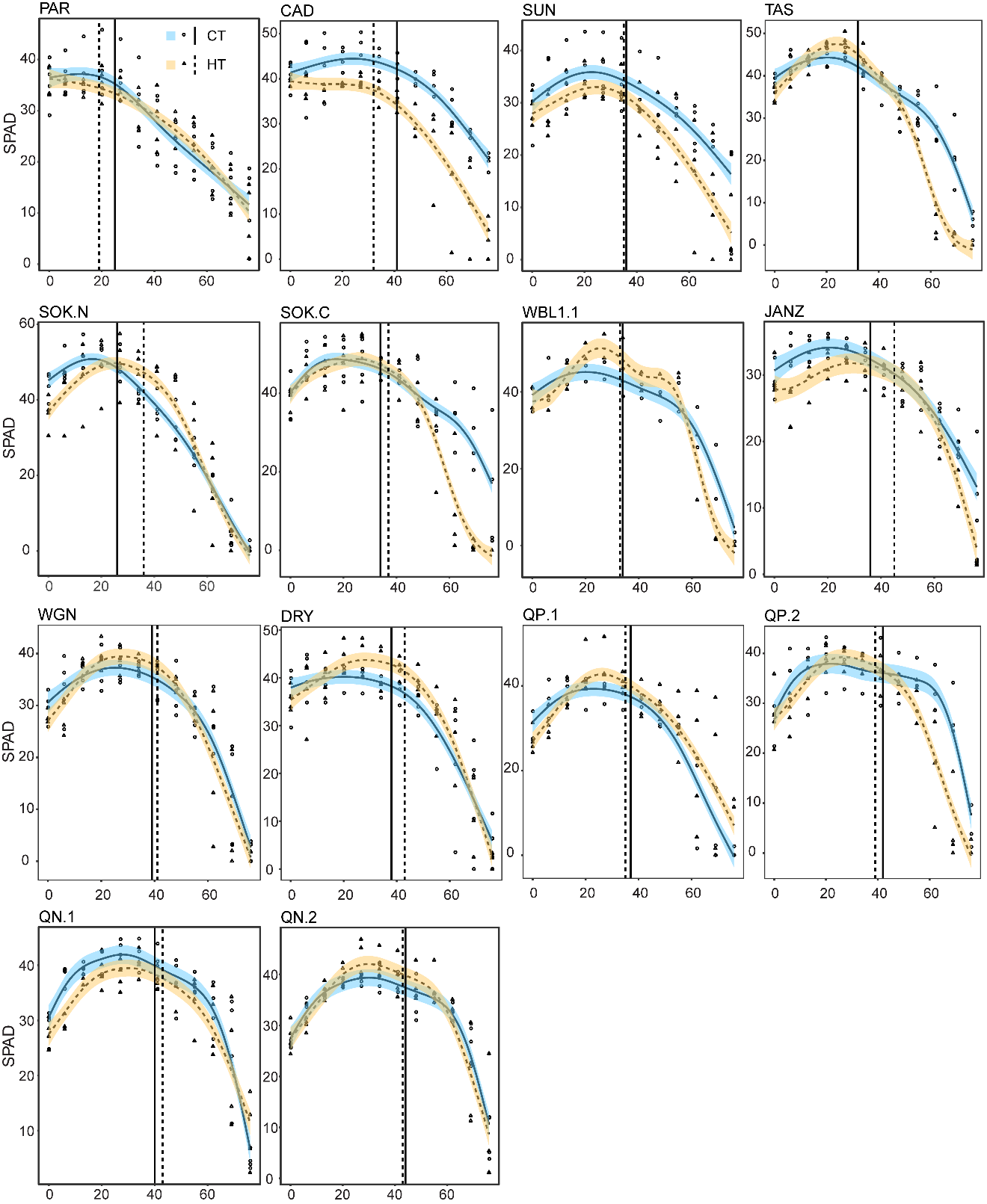


Fig S8. Generalised additive models (GAM) fitting for each genotype in Exp2. The vertical lines (solid, CT; dashed HT) indicate senescence onset (SenOnset). Smooth lines show the mean smooth model estimates, shading around the lines shows 95% confidence of the mean model estimate (yellow, CT; blue, HT).


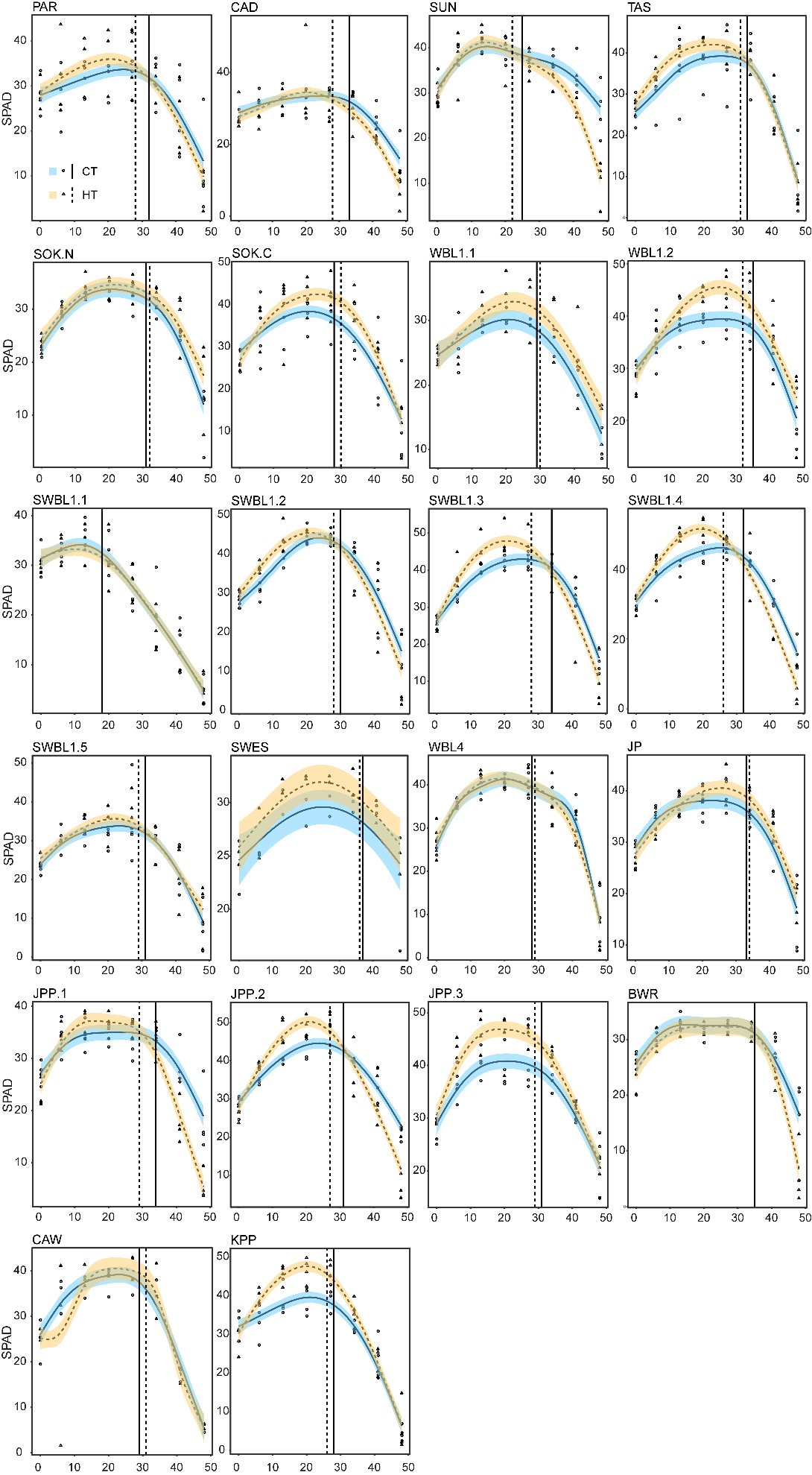


Fig S9. Generalised additive models (GAM) fitting for each genotype in Exp3. The vertical lines (solid, CT; dashed HT) indicate senescence onset (SenOnset). Smooth lines show the mean smooth model estimates, shading around the lines shows 95% confidence of the mean model estimate (yellow, CT; blue, HT).


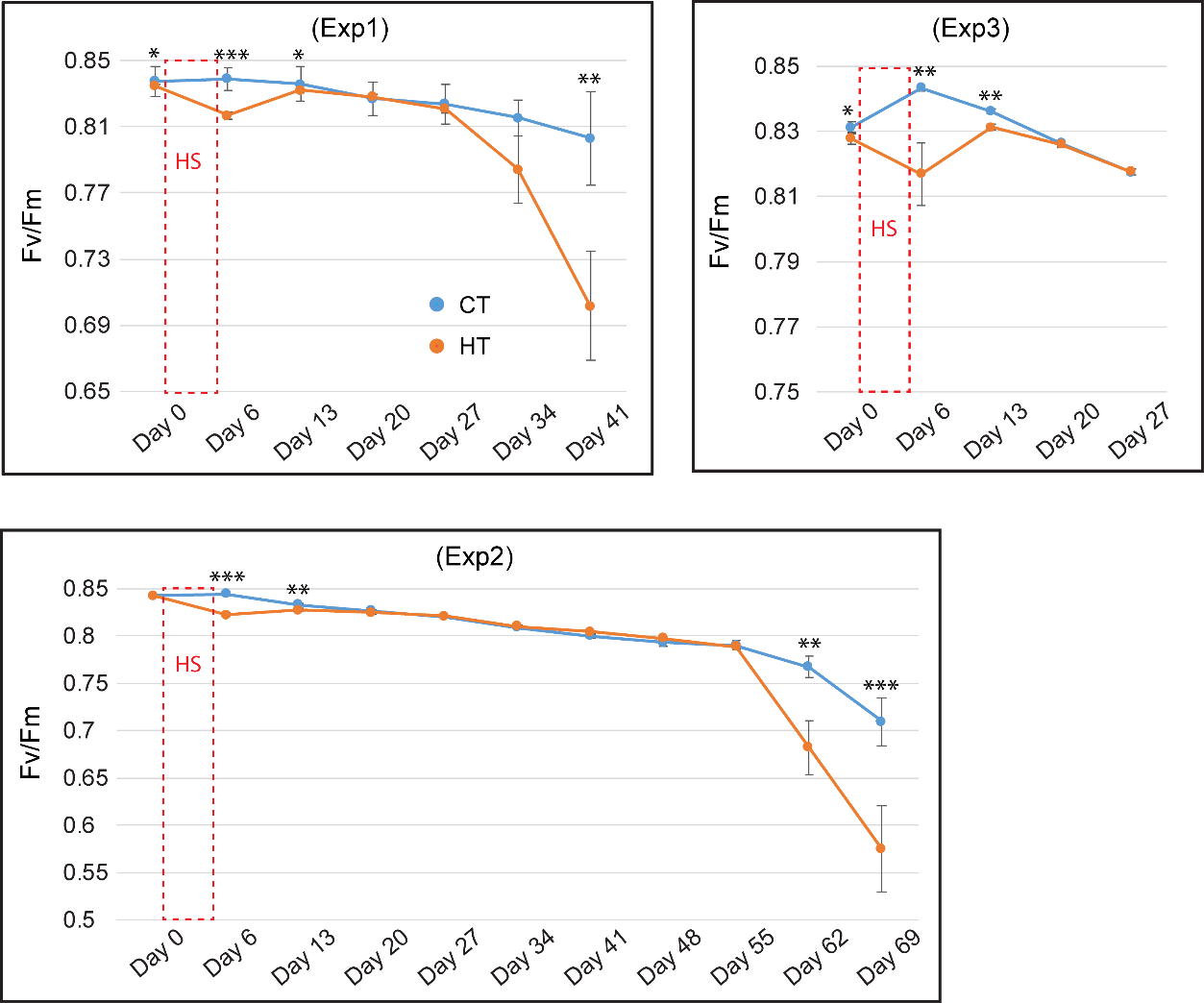


Fig S10. Comparing the heat (HT) and control (CT) effects on the dynamic change of Fv/Fm before (day 0), after (day 6) the 5-day HT treatment (day 1 - 5), and weekly intervals in Exp1 (a), Exp2 (b) and Exp3 (e). Significance level: P < 0.001 ***; P < 0.01 **; P < 0.05 *.
